# Validations of an alpha version of the E^3^ Forensic Speech Science System (E^3^FS^3^) core software tools

**DOI:** 10.1016/j.fsisyn.2022.100223

**Published:** 2022-03-07

**Authors:** Philip Weber, Ewald Enzinger, Beltrán Labrador, Alicia Lozano-Díez, Daniel Ramos, Joaquín González-Rodríguez, Geoffrey Stewart Morrison

**Affiliations:** a Forensic Data Science Laboratory, Aston University, Birmingham, UK; b Forensic Evaluation Ltd, Birmingham, UK; c Eduworks Corporation, Corvallis, OR, USA; d AUDIAS – Audio, Data Intelligence and Speech, Escuela Politécnica Superior, Universidad Autónoma de Madrid, Madrid, Spain

**Keywords:** Forensic voice comparison, Validation, Likelihood ratio, x-vector

## Abstract

This paper reports on validations of an alpha version of the E^3^ Forensic Speech Science System (E^3^FS^3^) core software tools. This is an open-code human-supervised-automatic forensic-voice-comparison system based on x-vectors extracted using a type of Deep Neural Network (DNN) known as a Residual Network (ResNet). A benchmark validation was conducted using training and test data (*forensic_eval_01*) that have previously been used to assess the performance of multiple other forensic-voice-comparison systems. Performance equalled that of the best-performing system with previously published results for the *forensic_eval_01* test set. The system was then validated using two different populations (male speakers of Australian English and female speakers of Australian English) under conditions reflecting those of a particular case to which it was to be applied. The conditions included three different sets of codecs applied to the questioned-speaker recordings (two mismatched with the set of codecs applied to the known-speaker recordings), and multiple different durations of questioned-speaker recordings. Validations were conducted and reported in accordance with the “Consensus on validation of forensic voice comparison”.

## 1 Introduction

There have been calls since the 1960s for forensic voice comparison to be validated under casework conditions (see [1] for a review). In recent years, researchers and practitioners have made substantial progress in promoting validation of human-supervised-automatic forensic-voice-comparison systems that output likelihood ratios. Lists of published papers including validations under forensically realistic conditions are included in [2] and [3]. These two lists have substantial overlap, and include papers published in *forensic_eval_01*, a 2016–2019 virtual special issue of the journal *Speech Communication* in which multiple different systems were validated using the same set of data that reflected the conditions of a real case.^1^ In 2019–2020 a consensus on validation of forensic voice comparison was developed. The published statement of consensus [4] had 13 authors and an additional 7 supporters.

The E^3^ Forensic Speech Science System (E^3^FS^3^) is being developed by the Forensic Data Science Laboratory at Aston University, with contributions from AUDIAS (Audio, Data Intelligence and Speech) at Universidad Autónoma de Madrid and from other research laboratories and multiple operational forensic laboratories. E^3^FS^3^ is designed for conducting both forensic-voice-comparison research and forensic-voice-comparison casework. The design of E^3^FS^3^ is informed by end-user needs assessments conducted with researchers and forensic practitioners at partner organizations including the Chilean Investigative Police (Policía de Investigaciones, PDI), the German Federal Criminal Police Office (Bundeskriminalamt, BKA), the Netherlands Forensic Institute (NFI), the Swedish National Forensic Centre (NFC), and the US Federal Bureau of Investigation (FBI). When complete, E^3^FS^3^ will include open-code software tools, data-collection protocols, databases (including those used for the present paper), standards and guidelines (including [4]), standard operating procedures, a library of validation reports (including the present paper), and training for practitioners. As each component of E^3^FS^3^ reaches the stage at which it is suitable for general release, it will be made available at http://e3fs3.forensic-voice-comparison.net/.

The core software tools of E^3^FS^3^ are based on state-of-the-art automatic-speaker-recognition technology, and are accompanied by detailed documentation explaining which algorithms were implemented and why they were chosen [5]. For maximum transparency, the software is written in Python (a popular free high-level programming language) and the code is extensively commented.^2^

The present paper describes the validation of an alpha version of the E^3^FS^3^ core software tools (hereinafter “E^3^FS^3^α”). The software tools are designed to be flexible and provide the user with various options and the ability to retrain models. In the present paper we describe the particular options and models that formed the system that was actually validated. The validations reported here are similar to validations that were performed prior to E^3^FS^3^α being used to compare the questioned-speaker and known-speaker recordings in a case.^3^ In the case, the speakers of interest on the questioned-speaker and known-speaker recordings were female speakers of Australian English, and there were multiple questioned-speaker recordings of different durations. In the context of the case, validations were conducted using recordings of female speakers of Australian English from which exactly the same number of feature vectors were extracted as from the questioned-speaker recordings in the case. For the validations reported in the present paper, we use recordings with a range of durations but not exactly the same number of extracted feature vectors as for the case. We performed three blocks of validations:

• a benchmark validation using the *forensic_eval_01* training and test data
• a series of validations reflecting the conditions of the case and a range of questioned-speaker-recording durations, but using recordings of male speakers rather than female speakers
• a series of validations reflecting the conditions of the case and a range of questioned-speaker-recording durations, and using recordings of female Australian English speakers

The benchmark validation allowed us to compare the performance of E^3^FS^3^α with that of other forensic-voice-comparison systems that had previously been validated on the same data. The validations using case-specific conditions but with non-case-specific male speakers were originally conducted so that if performance under those conditions had been poor we could have made modifications to the system to potentially improve performance before proceeding to validations on case-specific female speakers. The final validations should simply be validations of the performance of the system that was already selected for use in the case, one should not optimize the system using the final validation data since this would lead to overly optimistic final validation results. Performance on the male speakers was good, so, in actuality, we did not modify the system.

Below, we first describe E^3^FS^3^α, we then describe the benchmark validation and results followed by the case-specific validations and results. We end with discussion and conclusion.

We write assuming readers who are familiar with human-supervised-automatic forensic-voice-comparison systems to the level presented in [2], and who are familiar with validation of forensic-evaluation systems that output likelihood-ratio values to the level presented in [4].

When the core software tools are ready for general release at http://e3fs3.forensic-voice-comparison.net/, scripts to run the validations reported in the present paper will be also provided at that website. The data used for the validations reported in the present paper are already available at http://databases.forensic-voice-comparison.net/.

## 2 E^3^FS^3^ core software tools

### 2.1 System architecture

**Fig. 1:**
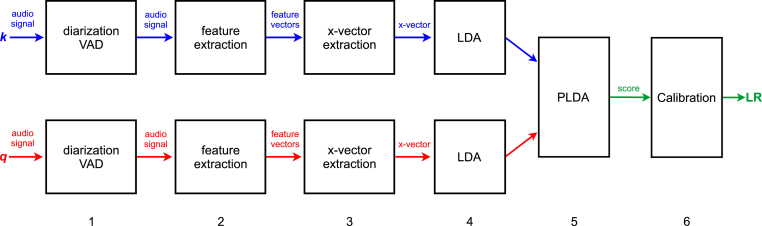
High-level architecture of E^3^FS^3^α.

The high-level architecture of E^3^FS^3^α is presented in Fig. 1. It consists of the following stages:

1. Speaker diarization and voice-activity detection (VAD)
2. Feature extraction
3. x-vector extraction
4. Dimension reduction and mismatch compensation using linear discriminant analysis (LDA)
5. Calculation of uncalibrated likelihood ratios (scores) using probabilistic linear discriminant analysis (PLDA)
6. Calibration

Data from the questioned-speaker recording and data from the known-speaker recording are processed in parallel through Stages 1–4. Stages 5 and 6 operate on data from pairs of recordings. Recordings used for training and validating the system (not shown in Fig. 1) are processed in the same manner as the data from the questioned-speaker and known-speaker recordings. Terminologically: Stage 1 (diarization and VAD) are key parts of preprocessing; Stage 3 (x-vector extraction) constitutes the frontend model; and Stages 4–6 (LDA, PLDA, and calibration) constitute the backend models.

We briefly describe each stage of the system in its own subsection below. Since the particular x-vector-extraction stage used is different from that presented in [2] and likely to be less familiar for many readers, we describe this stage in somewhat greater detail.

### 2.2 Diarization and VAD

E^3^FS^3^ will include a diarization tool based on the VBx algorithm, which had the best performance in the DIHARD’19 diarization challenge [[6], [7], [8], [9]]; however, all data that were used for training and validation in the context of the present paper were supplied already diarized, so this part of the system was not validated as part of the E^3^FS^3^α validation.

E^3^FS3α performs VAD using the rVAD-fast algorithm [10]. This is an unsupervised method, which has the advantage of not requiring labelled training data. It can achieve a similar level of performance to supervised methods when the latter are trained and tested on the same conditions [10]. The algorithm applies two noise-removal processes: The first process attempts to remove transient noises, and the second process attempts to remove background noise. The next stage in the algorithm searches for voiced speech sounds using a spectral flatness detector (which is faster than fundamental-frequency detection, which used in the original rVAD algorithm). In order to also include voiceless sounds, the sections of the recording identified as containing voiced sounds are extended by 60 frames (600 ms) both before and after. The final stage uses heuristics based on the energy differences between frames to select frames deemed to be speech.

### 2.3 Feature extraction

Until recently, mel-frequency cepstral coefficients (MFCCs) [11] were the most commonly used features for automatic-speaker-recognition systems, but log-mel-filterbank features have been found to be more effective for x-vector systems [8], [12], [13].

E^3^FS3α uses the implementation of log mel filterbanks described in [14] §3.1.5. A 25 ms duration Hamming window is used. All the training and validation data were either originally 8 kHz sampling rate 16 bit quantization or were resampled to 8 kHz sampling rate 16 bit quantization, hence 25 ms is equivalent to 200 samples. The power spectrum within the window is calculated using a 512-point fast Fourier transform. The filterbank consists of 40 filters, equally spaced on the mel frequency scale, that together cover the frequency range 0–4 kHz. Each filter has a 50% overlap with each of its neighbours. The window is advanced in steps of 10 ms (80 samples). There is therefore a 60% overlap between adjacent frames. The window is repeatedly advanced until feature vectors have been extracted from all the speech of the speaker of interest on a recording. A series of consecutive feature vectors we will refer to as a “feature matrix”.

### 2.4 x-vector extraction

**Fig. 2:**
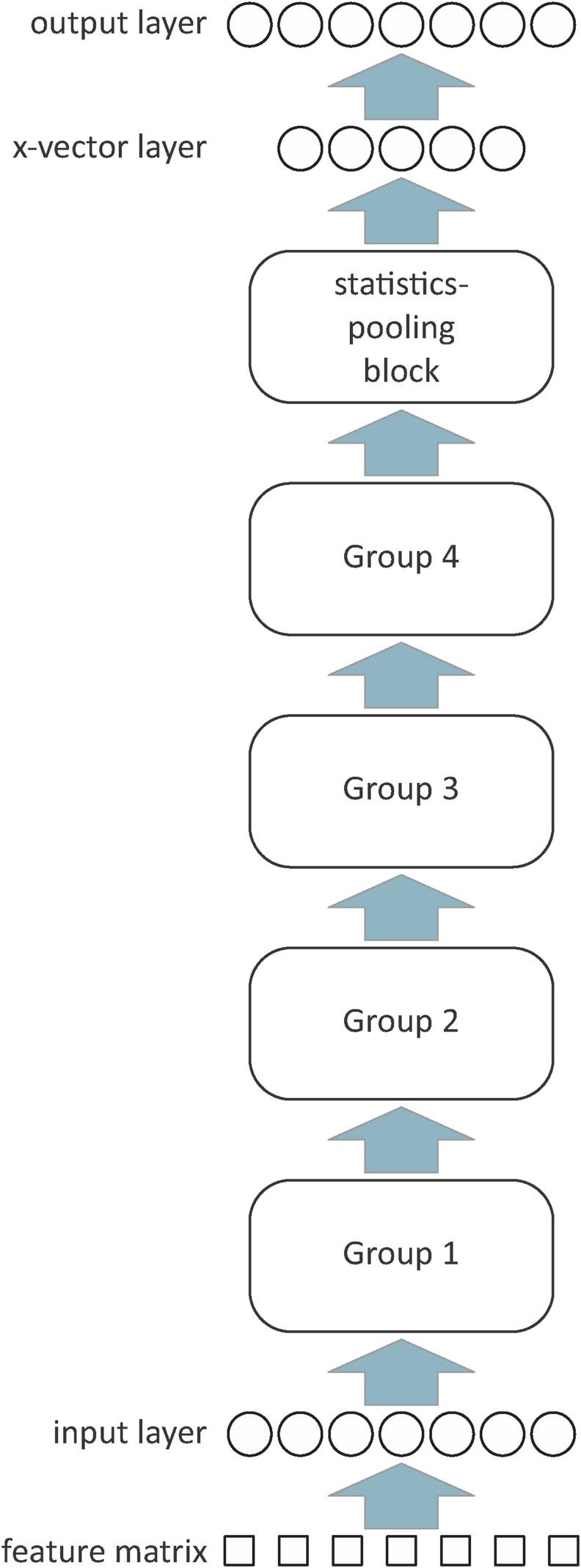
Simplified schematic of the architecture of the ResNet used for extracting x-vectors.

E^3^FS^3^α extracts x-vectors using a type of deep neural network (DNN) called a Residual Network (ResNet; [15]). In particular, it uses a variant of the ResNet34 architecture described in [16] and [17]. The ResNet consists of a series of “groups”, each group consists of a series of “blocks”, and each block consists of a series of “layers”. The architecture of the ResNet is summarized in Fig. 2, and the sizes of the dimensions of its structures and substructures are given in Table 1.

**Table 1:** Sizes of the dimensions of the structures and substructures of the ResNet used for extracting x-vectors.

Structure	Substructure	Dimensions		
		time *T*	frequency *F*	channels *C*
Feature vectors	–	400	40	1
Input layer	–	400	20	16
Group 1	3 blocks	400	20	16
Group 2	4 blocks	200	10	32
Group 3	6 blocks	100	5	64
Group 4	3 blocks	100	5	128
Statistics-pooling block	Layer 1	100	1	128
	Channel-attention layer	1	1	128
	Layer 2	100	1	1
	Layer 3	1	1	128
x-vector layer	–	1	1	512
Output layer	–	1	1	Number of training speakers

**Fig. 3:**
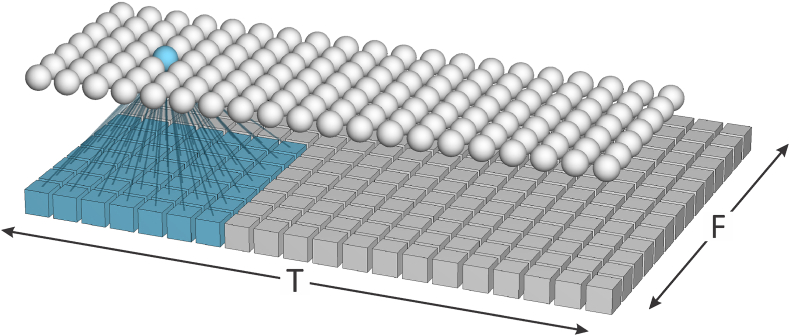
Simplified schematic of the feature vectors and the input layer of the ResNet used for extracting x-vectors. Only one channel is shown.

Each input-layer node of the ResNet receives input from a square “patch” of feature values which covers 7 time steps by 7 frequency steps of the feature matrix, see Fig. 3. The “stride” in the time dimension is 1 and the stride in the feature dimension is 2; hence, the length of the input-layer rows equals the number of feature vectors entered from a recording, *T* (which for training is 400), and the length of the input-layer columns is half the length of each feature vector, i.e., since the length of a feature vector is 40, the length of an input-layer column is 20. The same “kernel” (set of connection weights between each node in the input layer and its corresponding patch of feature values) is used for all input-layer nodes (the activations of the nodes of the input layer are the result of convolving the kernel with the feature matrix). Additional kernels are created by initializing the connections with different sets of weights. Each additional kernel is used for all nodes in an additional input layer that is parallel to the first input layer. Each parallel input layer creates a “channel”. The number of channels, *C*, for the input layer is 16.

**Fig. 4:**
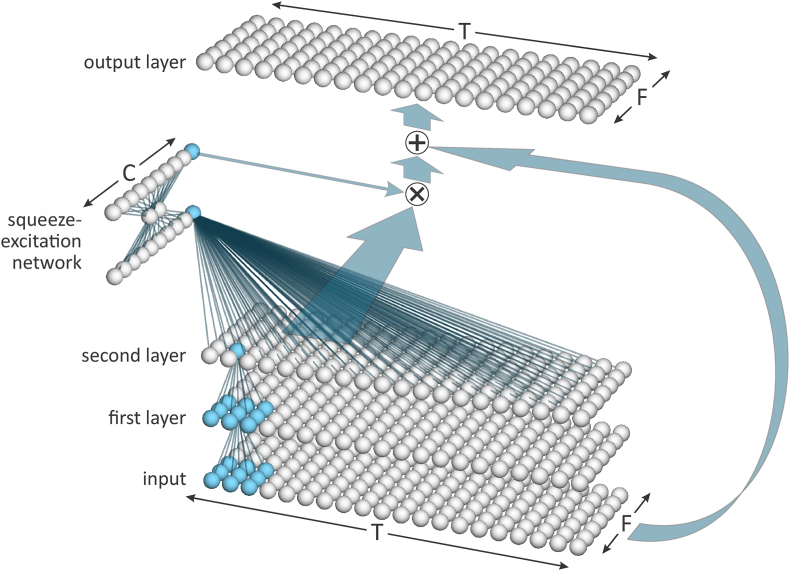
Simplified schematic of the architecture of a block within the ResNet used for extracting x-vectors. Only one channel is shown.

The input layer is followed by a series of 4 groups, see Fig. 2. Each group consists of multiple blocks. Fig. 4 provides a simplified schematic of the architecture of a block. The first two layers of each block in each group use kernels that cover 3 time steps by 3 frequency steps of the output from the immediately preceding layer of each channel, i.e., a 3 × 3 kernel for each of *C* channels. In Groups 2 and 3, the stride for the first layer of the first block is 2 for both the time and frequency dimensions (hence the size of each dimension is halved). For the first two layers of all other blocks in all groups, and for the second layer of the first block in each of Group 2 and 3, the stride is 1 in each dimension (hence the size of the dimensions is unchanged). For the first layer of the first block of each of Groups 2 through 4, two kernels are applied to the output of the previous group. This results in a doubling of the number of channels.

After the first two layers of each block in Groups 1 through 4, there is a one-dimensional “squeeze-excitation network”. Each node in the input layer of this network calculates the mean value of all the nodes in the previous layer belonging to a single channel. The network then has a bottleneck layer and an output layer. The output layer has the same number of nodes as the input layer, i.e., one per channel. The activations of the nodes in the output layer are used to weight the channels relative to one another. This focuses “attention” on the channels that are more useful for distinguishing speakers from one another [18].

For each channel, the output of a block is the elementwise sum of the channel-weighted output of the block’s second layer and the original input to the block. If there is a difference in the number of time or frequency steps or the number of channels between the previous block and the current block, in order to be able to perform the elementwise sum, the input to the block is processed through a set of kernels that alter its dimensions to match those of the current block. This set of kernels is independent of other sets of kernels. The addition of the input to a block to what would otherwise be its output is the “residual” that gives ResNets their name.

**Fig. 5:**
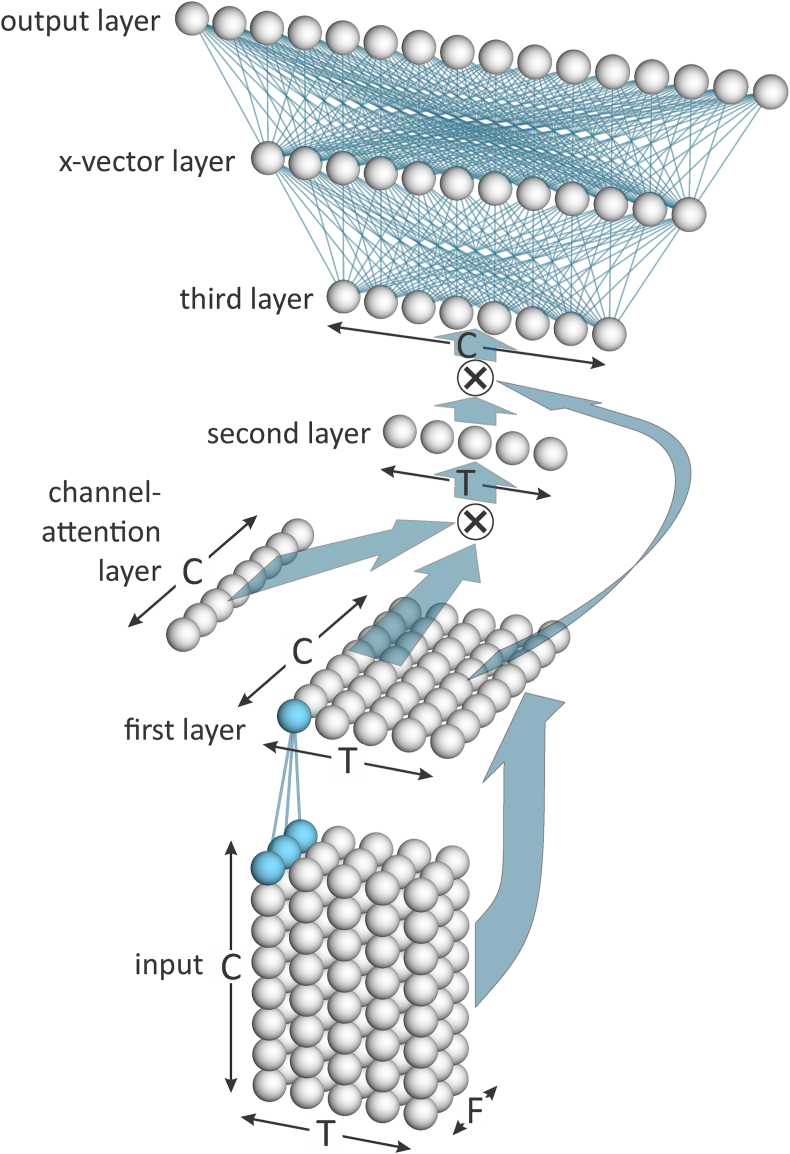
Simplified schematic of the final stages of the ResNet used for extracting x-vectors. In this figure “ × ” indicates matrix multiplication.

The final stages of the ResNet consist of a statistics-pooling block, an x-vector layer, and an output layer, see Fig. 5.

The first layer of the statistics-pooling block collapses the frequency dimension by calculating the mean of the column of frequency values corresponding to each time step of the immediately preceding layer. This is done separately for each channel, and the result is treated as a two-dimensional time by channel (*T* × *C*) layer.

After the first layer of the statistic-pooling block, similar to the squeeze-excitation networks in earlier blocks, there is a one-dimensional “channel-attention layer” which is the same length at the number of channels. Unlike the squeeze-excitation networks, the channel-attention layer is a single layer and is only connected to a higher layer, it does not have input from a lower layer. The activations of the nodes in the channel-attention layer are therefore learned during training, and thereafter are fixed. The activations of the nodes in the channel-attention layer are used to weight the channels of the statistics-pooling block’s first layer. The result, the second layer, is a one-dimensional layer that is the same length as the number of time steps, and in which the activation of each node is a function of the weighted sum of the activations of the first layer’s nodes at one of its time steps (before being weighted by the channel-attention layer, a non-linear function is applied to the activations of each of the nodes in the first layer). The activations of the nodes in the second layer are then used to weight the time steps of the statistic-pooling block’s first layer. The result, the third layer, is a one-dimensional layer that is the same length as the number of channels, and in which the activation of each node is a function of the weighted sum of the activations of the first layer’s nodes for one of its channels. The third layer has combined information from across both time and frequency.

The third layer of the statistics-pooling block is fully connected to the x-vector layer, which has 512 nodes, and the x-vector layer is fully connected to the output layer. The output layer has one node for each speaker in the training data. An angular-margin softmax function (AMSoftmax [19]) is applied to the output layer.

We trained the ResNet using approximately 1M recordings total from approximately 6k speakers from the VoxCeleb2 database [20]. Training was conducted using 200 epochs of the Adam variant of the stochastic gradient descent optimization algorithm [21]. The learning rate started at 0.001 and every 10 epochs was decreased by 5%. For training, 400 feature vectors were presented at a time (*T* = 400). To prevent overfitting on the training data, batch normalization was applied to each of the first two layers of each block in Groups 1 through 4 (see [22] [23] [24]). Batches of data were used to train the system. Each batch consisted of a set of 200 recordings, one recording from each of 200 speakers. After every batch, batch normalization adjusted the activations of the nodes so that, over the whole batch, the mean and standard deviation of the activations over all the nodes of each *T* × *F* layer were 0 and 1 respectively.

For extraction of x-vectors, recordings longer (or shorter) than 400 feature vectors can be presented to the ResNet. Since the same kernels are used for each node in the input layer, the number of nodes in the rows of the input layer can be increased (or decreased) to accommodate the number of feature vectors, and the kernels simply repeated for each node – no retraining is needed to accommodate the different number of feature vectors. Because the first two layers of each block in Groups 1–4, also use kernels, the number of time steps in higher layers can likewise be increased (or decreased) without the need for retraining. The statistics-pooling block collapses the time dimension (and the frequency dimension) so that the three final one-dimensional layers of the ResNet have the same numbers of nodes irrespective of the number of feature vectors and irrespective of the numbers of time steps in earlier layers.

### 2.5 LDA

From Stage 6 onward, the data used for training or adapting the backend models should be representative of the relevant population for the case and should reflect the conditions of the questioned-speaker and known-speaker recordings for the case, including any mismatch in conditions between the questioned-speaker and known-speaker recordings.

Although referred to in the automatic-speaker-recognition literature as LDA, Stage 6 is actually the use of linear discriminant functions (LDFs). LDFs are used for mismatch-compensation and to reduce the number of dimensions of the x-vector. E^3^FS^3^α trains the LDFs using the algorithm described in [25] §4.3, and reduces the x-vectors from 512 to 120 dimensions. In addition to using x-vectors from recordings that actually reflect the population and conditions for the case (in-domain data), using the correlation-alignment algorithm (CORAL) [12], [26], x-vectors from a large number of non-case-specific recordings of a large number of speakers (out-of-domain data) are adapted to simulate this population and these conditions. E^3^FS^3^α uses the CORAL algorithm described in [12], which linearly shifts and scales the out-of-domain data so that their total covariance matrix (within-speaker plus between-speaker covariance matrix) matches that estimated from the in-domain data.

As out-of-domain data for CORAL, we used approximately 30k recordings total from approximately 2.7k speakers from the SRE2018 test set [27].

### 2.6 PLDA

E^3^FS^3^α implements the two-covariance variant of PLDA described in [28]. The training algorithm is iterative. 100 iterations are used. PLDA calculates a common-source likelihood ratio [29], but because of the large number of parameter values that have to be estimated to fit the model in the 120 dimension space, the output is treated as an uncalibrated log likelihood ratio (also known as a “score”).^4^

Prior to training the PLDA model, to better fit the assumptions of the model, the training data are centered, whitened (i.e., rotated and scaled so that for the entire training set the variance in each dimension is 1 and the covariance between dimensions is 0), then scaled to unit length in the Euclidian multidimensional space [33]. The x-vectors that will be used for calibration and validation are transformed using the centering and whitening functions derived from the training data, and then scaled to unit length.

### 2.7 Calibration

E^3^FS^3^α uses logistic regression to convert the uncalibrated log likelihood ratios to calibrated log likelihood ratios. The calibration model is trained using a regularized version of the conjugate-gradient method [34], [35], with a regularization weight equivalent to 1 pseudo-speaker [36].^5^ This regularization reduces the probability of overstating the strength of evidence in either direction.

To maximize use of available case-relevant data, and to avoid training and testing on the same data, the calibration model is trained using leave-one-speaker-out/leave-two-speakers-out cross-validation, see [4] §2.5.4.

## 3 Benchmark validation

### 3.1 Data

The *forensic_eval_01* benchmark dataset and validation protocols are described in [37] and [38]. The speakers are male Australian-English speakers. The questioned-speaker condition reflects a 46 s long landline-telephone call, with background babble noise, saved using lossy compression (G.723.1). The known-speaker condition reflects a 126 s long interview recorded in a reverberant room, with background ventilation-system noise. The durations just stated refer to the amount of speech of the speaker of interest after semi-manual diarization but before applying VAD. The questioned-speaker-condition and known-speaker-condition recordings were recorded on different occasions separated by approximately a week or more. Each speaker in the calibration/validation set was recorded on at least two occasions. The calibration/validation set consists of a total of 223 recordings from 61 speakers, 61 in questioned-speaker condition and 162 in known-speaker condition, allowing for the construction of 111 same-speaker pairs of recordings and 6,720 different-speaker pairs of recordings (from 3,660 pairs of speakers). The dataset also includes a training set consisting of a total of 423 recordings from 105 speakers (191 recordings in questioned-speaker condition and 232 in known-speaker condition).

The *forensic_eval_01* training set was used to train the LDA and PLDA models (along with the previously-mentioned out-of-domain data that was adapted using CORAL, see §2.5). The *forensic_eval_01* calibration/validation set was used for leave-one-speaker-out/leave-two-speakers-out cross-validated training of the calibration model.

### 3.2 Results and discussion

**Fig. 6:**
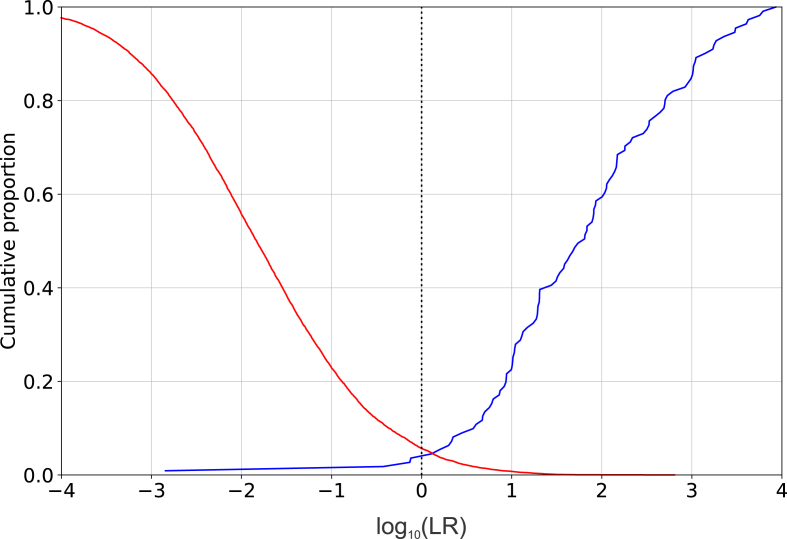
Tippett plot of the results of validating E^3^FS^3^α using the *forensic_eval_01* data.

A Tippett plot showing validation results for E^3^FS^3^α using the *forensic_eval_01* data is presented in Fig. 6. Table 2 presents the *C*_llr_ results from the best-performing version of each system validated in the *Speech Communication* virtual special issue [39], plus the *C*_llr_ result from the validation of E^3^FS^3^α.

**Table 2:**
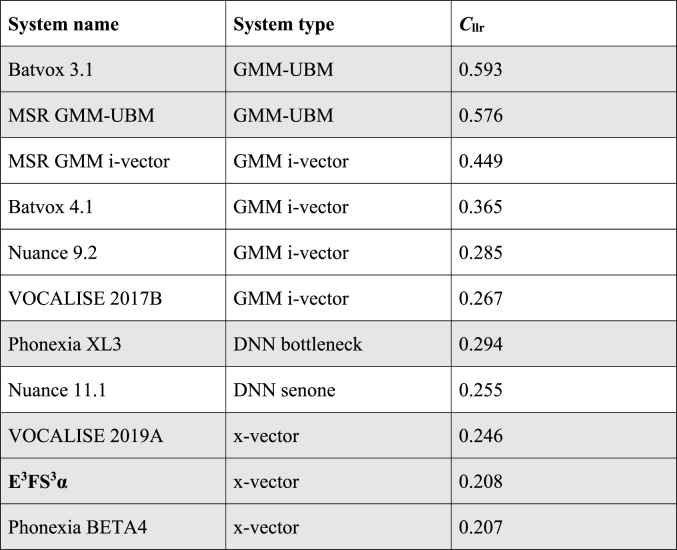
*C*_llr_ values from the best-performing version of each system validated in the *Speech Communication* virtual special issue, plus the *C*_llr_ result from E^3^FS^3^α, each validated using the *for ensic_eval_01* data. Alternating background shading groups types of systems.

In the Tippett plot (Fig. 6), the same-speaker and different-speakers curves have relatively shallow slopes, indicating good performance, and they cross near a log-likelihood-ratio value of 0, indicating good calibration. The Tippett plot indicates that the validation results for E^3^FS^3^α would support likelihood-ratio values into the thousands in favour of the same-speaker hypothesis and into the tens of thousands in favour of the different-speaker hypothesis (log_10_ likelihood ratios beyond +3 and −4 respectively), but not values far beyond these ranges (see [4] §2.11).

The *C*_llr_ value for E^3^FS^3^α was 0.208. The lower the *C*_llr_ value, the better the performance of the system. In terms of *C*_llr_, E^3^FS^3^α performed equally as well as the best-performing system from the virtual special issue, Phonexia SID-BETA4 [40].

Based on these benchmark-validation results, we were happy with the performance of E^3^FS^3^α, and proceeded to test it under conditions that reflected those of the case to which it was to be immediately applied.

## 4 Case-specific validations

### 4.1 Casework conditions

The forensic case involved a number of questioned-speaker recordings in which the speaker of interest was a female speaker of Australian English, and a number of known-speaker recordings in which the speaker of interest was a female speaker of Australian English. All recordings were of telephone calls made from a mobile telephone to a call centre and were recorded at the call centre. Multiple call centres were involved, and multiple calls were recorded at each call centre.

All recordings were converted to PCM, 8 kHz sampling rate, 16 bit quantization using FFmpeg software,^6^ and the results were checked using MediaInfo^7^ (the condition characterization and format conversion tools of E^3^FS^3^ were still under development). Each recording was manually diarized using Audacity^8^ (the manual diarization tool of E^3^FS^3^ was still under development), and reference headphones (AKG K701 or K702) for listening. The questioned-speaker recordings were diarized by one practitioner and the known-speaker recordings were diarized by a second practitioner. Sections of speech of the speaker of interest were excluded if they:

• overlapped with the speech of the interlocutor or with transient noises;
• were obviously distorted or muffled; or
• had raised vocal effort, non-modal phonation, or appeared to reflect an agitated emotional stage.

After initial diarization, the original diarizer checked the results and made corrections as needed. This included checking the labels to make sure that only speech of the speaker of interest was marked as such. It also included listening to every section that had been marked as speech of the speaker of interest. A third practitioner then checked the diarization results. The third practitioner was instructed to flag any errors they found including:

• sections with missing labels;
• noise labelled as speech;
• speech labelled as noise;
• speech labelled as belonging to the speaker of interest but potentially belonging to another speaker;
• speech labelled as belonging to another speaker but potentially belonging to the speaker of interest;
• speech labelled as belonging to the speaker of interest but including substantial distortion or transient noises;
• beginning or end markers that appeared to be misplaced.

The third practitioner then discussed with the original diarizer any errors the third partitioner had flagged, with both practitioners able to see and listen to the relevant sections of the recordings. If both immediately agreed on the appropriate correction to be made, the original diarizer made the correction. If they did not immediately agree on the appropriate correction to be made, the original diarizer deleted the markers and the label for the section.

These procedures were adopted in order to reduce the potential for cognitive bias: Since neither of the first two practitioners listened in detail to the speech on both the questioned-speaker recording and the known-speaker recording, the strength-of-evidence conclusions could not be affected by subjective judgements related to their perceptions of the speech on the recordings. The third practitioner waited 2 months between finishing checking the diarization of the questioned-speaker recordings and starting checking the diarization of the known-speaker recordings. This time interval was intended to be sufficient to prevent the third practitioner from perceptually comparing the speech on the questioned-speaker and known-speaker recordings. The third practitioner was instructed not to attempt to perceptually compare the questioned-speaker and known-speaker recordings, and not to discuss with either of the first two practitioners any perceptions or opinions the third practitioner may have inadvertently formed. The third practitioner did not play any role in subsequent processing of the case data or in writing or reviewing the casework report.

Most of the known-speaker recordings had the same format:

• **μ-law + G.723.1:** 2 channels, μ-law (commonly used in landline-telephone systems), 8 kHz sampling rate, 8 bit quantization. Enquiries made through the instructing party to the suppliers of the recordings revealed that these recordings had previously been saved using G.723.1 compression (commonly used for VoIP).

The speaker of interest was on one channel and the interlocutor on another, and over the whole or substantial portions of each recording the speaker of interest’s channel had no apparent background noise (measured signal-to-noise ratios were above 60 dB). A group of recordings consisting of the 7 longest recordings in this condition (each having at least ∼120 s net speech) was used for comparison with each of the questioned-speaker recordings. The other recordings in this condition had less than ∼90 s net speech.^9^ The latter were used to conduct an additional set of validations (results of which are not reported in the present paper): The group of the longest recordings was compared with each of the shorter recordings (these are same-speaker comparisons), and with recordings of other female Australian English speakers in the same condition and of the same duration (these are different speaker comparison). The remaining known-speaker recordings had other conditions, including poorer conditions, and were not used.

The same number of feature vectors (corresponding to ∼120 s net speech) were extracted from each recording in the group of the 7 longest known-speaker recordings. An x-vector was independently extracted from each recording in this group, and the mean vector of these x-vectors used as input to the backend models.

On initial screening, some questioned-speaker recordings with poor recording conditions were excluded from use on the grounds that they were a priori unlikely to lead to log likelihood ratios far from 0 in either direction. The remaining questioned-speaker recordings each had one of three formats:

• **GSM 06.10:** 1 channel, GSM 06.10 (commonly used in mobile-telephone systems), 8 kHz sampling rate, 13 kb/s bit rate. Enquiries made through the instructing party to the suppliers of the recordings revealed that these recordings had not previously been saved in another format.
• **μ-law + G.729a:** 1 channel, μ-law, 8 kHz sampling rate, 8 bit quantization. Enquiries made through the instructing party to the suppliers of the recordings revealed that these recordings had previously been saved using G.729a compression (commonly used for VoIP).
• **μ-law + G.723.1:** The same as the known-speaker condition.

Over the whole or substantial portions of each recording there was no apparent background noise (measured signal-to-noise ratios were above 60 dB). The questioned-speaker recordings had a range of different durations leading to a range of different numbers of feature vectors extracted from each recording. For the case, a different validation was conducted for each different number of feature vectors. For the validations reported in the present paper, we do not use exactly the same number of extracted feature vectors. Instead, we use the numbers of feature vectors given in Table 3.

**Table 3:** Numbers of feature vectors and corresponding net-speech durations of the questioned-speaker-condition recordings used for the case-specific validations reported in the present paper.

Number of feature vectors extracted	Net-speech duration (s)
500	5
1,000	10
1,500	15
2,000	20
3,000	30
4,500	45
6,000	60
9,000	90
12,000	120
18,000	180

### 4.2 Data

Training and calibration/validation data were taken from the AusEng 500+ database [41]. The data-collection protocol for this database is described in [42].

The database includes recordings of 169 male Australian-English speakers who were recorded in at least two recording sessions. Each session was separated by approximately a week or more. 43 male speakers had two sessions, 107 had three, and 19 had more (5 had four, 4 had five, 4 had six, 4 had seven, and 2 had eight). The database also includes recordings of 233 female Australian-English speakers who were recorded in at least two recording sessions. 69 female speakers had two sessions, 159 had three, and 5 had more (2 had four, 1 had five, and 2 had seven).

In each session, the speaker completed multiple speaking tasks. We concatenated and used recordings of two tasks: telephone conversation, and information exchange over the telephone. The lead forensic practitioner considered the combination of these two tasks to be sufficiently reflective of the speaking styles in the diarized portions of the questioned-speaker and known-speaker recordings (this is a subjective judgement). The recordings were high-quality audio recordings (direct microphone recordings, not recordings of a signal transmitted through a telephone system). One speaker was recorded on each channel. Prior to release of the database, the channels had been separated, an automated VAD process had been applied to find the sections of each recording that potentially contained speech, and those sections had been manually checked and corrected as needed. The sections within a recording had then been concatenated and saved as PCM, 16 kHz sampling rate, 16 bit quantization. As part of the concatenation process, ramp-up and ramp-down windows were used to avoid introducing discontinuities.

E^3^FS3 includes a condition-simulation tool. Using this tool and the AusEng 500+ recordings, we simulated three different sets of conditions. In order to simulate each condition, a chain of transformations was applied:^10^

**GSM 06.10:** PCM, 16 kHz, 16 bits → PCM, 8 kHz, 16 bits → AMR codec to simulate mobile telephone transmission → G.711 a-law codec to simulate landline telephone transmission → GSM 06.10, 8 kHz, 13 kb/s codec → PCM, 8 kHz, 16 bits
**μ-law + G.729a:** PCM, 16 kHz, 16 bits → PCM, 8 kHz, 16 bits → AMR codec to simulate mobile telephone transmission → G.711 a-law codec to simulate landline telephone transmission → G.729a codec → G.711 μ-law, 8 kHz, 8 bits, codec → PCM, 8 kHz, 16 bits
**μ-law + G.723.1: PCM, 16 kHz, 16 bits → PCM, 8 kHz, 16 bits → AMR codec to simulate mobile telephone transmission → G.711 a-law codec to simulate landline telephone transmission → G.723.1 codec → G.711 μ-law, 8 kHz, 8 bits, codec → PCM, 8 kHz, 16 bits.**

The AMR-NB codec for simulation of GSM mobile telephone transmission uses the reference implementation for Adaptive Multi-Rate speech traffic [43]. The AMR codec has 8 encoding rates. For each recording, one of the rates was randomly selected from a uniform distribution. G.711 a-law and μ-law landline encoding uses the reference implementation [44]. The GSM 06.10 codec for GSM mobile telephone transmission uses the reference implementation [45]. The G.723.1 codec for VoIP compression uses the reference implementation [46]. The G.723.1 codec has 2 encoding rates. For each recording, one of the rates was randomly selected from a uniform distribution. The G.729a codec for VoIP compression uses the reference implementation [47].

Recordings from each speaker’s second and later recording sessions were used to create known-speaker-condition recordings. From each known-speaker-condition recording, a set of 12,000 contiguous feature vectors was randomly selected and the remainder discarded. For each recording, the start of the contiguous set of feature vectors was randomly selected from a distribution that was uniform over the first to the last-minus-12,000th feature vector. An x-vector was independently extracted from each known-speaker recording of each speaker. For each speaker who had more than one known-speaker-condition recording, the mean vector of their x-vectors was used as input to the backend models.

Recordings from each speaker’s first recording session were used to create questioned-speaker-condition recordings. From each questioned-speaker-condition recording, using the same procedure as described above for the known-speaker-condition recordings, sets of contiguous feature vectors were randomly selected and the remainder discarded. A different set of feature vectors was extracted corresponding to each net speech duration listed in Table 3.

The within-speaker variance, calculated using the known-speaker-condition mean vectors and the questioned-speaker-condition individual vectors, will be less than if the variance of the known-speaker-condition individual vectors had been used, and the more individual vectors that contribute to each known-speaker-condition mean vector the smaller the variance.^11^ For most speakers, the number of known-speaker-condition recordings per speaker was 2 (for some it was less, and only for a few was it more). The calculated within-speaker variance for the validation data will therefore be greater than if 7 individual known-speaker-condition vectors had been used to calculate each mean vector (in the case, 7 vectors were used to calculate the known-speaker mean vector). The smaller the within-speaker variance compared to the between-speaker variance, the further the log likelihood ratios can be away in both directions from the neutral value of 0. The validation results will therefore underestimate the performance of the system compared to if 7 individual vectors had been used. This is a limitation of the available data, but produces a conservative set of validation results.

For male speakers: 91 of the speakers were randomly selected and their x-vectors were used for training the LDA and PLDA models. x-vectors from the remaining 78 speakers were used for leave-one-speaker-out/leave-two-speakers-out cross-validated training of the calibration model. For each questioned-speaker condition including each duration, this resulted in 78 likelihood-ratio values from same-speaker comparisons and 6,006 likelihood-ratio values from different-speaker comparisons.

For female speakers: 125 of the speakers were randomly selected and their x-vectors were used for training the LDA and PLDA models. x-vectors from the remaining 108 speakers were used for leave-one-speaker-out/leave-two-speakers-out cross-validated training of the calibration model. For each questioned-speaker condition including each duration, this resulted in 108 likelihood-ratio values from same-speaker comparisons and 11,556 likelihood-ratio values from different-speaker comparisons.

## 5 Results and discussion

**Fig. 7:**
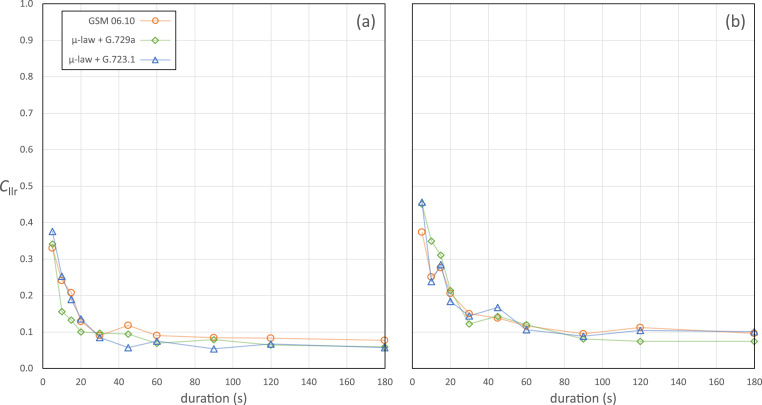
*C*_llr_ values for case-specific conditions. (a) Male speakers. (b) Female speakers.

Table 4 and Table 5 present the *C*_llr_ values for the validation results from the case-specific conditions for male and female speakers respectively. Fig. 7 provides a graphical representation of the relationship between questioned-speaker conditions, including net-speech duration, and *C*_llr_ values.

**Table 4:** *C*_llr_ values for case-specific conditions – male speakers.

Questioned-speaker net-speech duration (s)	Questioned-speaker condition		
	GSM 06.10	μ-law + G.729a	μ-law + G.723.1
5	0.330	0.341	0.376
10	0.241	0.156	0.253
15	0.208	0.133	0.189
20	0.129	0.100	0.136
30	0.090	0.097	0.085
45	0.118	0.094	0.057
60	0.090	0.069	0.075
90	0.084	0.079	0.054
120	0.083	0.064	0.067
180	0.077	0.059	0.057

**Table 5:** *C*_llr_ values for case-specific conditions – female speakers.

Questioned-speaker net-speech duration (s)	Questioned-speaker condition		
	GSM 06.10	μ-law + G.729a	μ-law + G.723.1
5	0.374	0.451	0.456
10	0.251	0.349	0.238
15	0.276	0.311	0.285
20	0.206	0.214	0.184
30	0.150	0.122	0.144
45	0.138	0.143	0.167
60	0.117	0.120	0.106
90	0.095	0.081	0.088
120	0.112	0.074	0.104
180	0.096	0.074	0.101

We can make the following observations on the results of the case-specific validations:

• Overall, *C*_llr_ values were substantially lower than those that we would have expected from earlier generations of technology (i-vector or GMM-UBM).
• Male speakers with a questioned-speaker net-speech duration of 45 s are the most similar population and duration to those of *forensic_eval_01. C*_llr_ values for this case-specific population and duration combination were substantially lower than that for *forensic_eval_01*: 0.118, 0.094, or 0.057 depending on the questioned-speaker condition (GSM 06.10, μ-law + G.729a, or μ-law + G.723.1 respectively), compared to 0.208 for *forensic_eval_01.* This is likely due to the case-specific conditions not being as poor as those of *forensic_eval_01*, which were based on a different case.
• *C*_llr_ values for male speakers were consistently lower than for female speakers. Given current information, we cannot assess whether the difference is due to the larger amount of validation data available for female speakers (a larger dataset may include more speakers who are similar to other speakers in the dataset), or whether it is due to a bias in E^3^FS^3^ training that results in better performance on males, or whether it is due to an intrinsic difference between properties of male and female speakers' speech. These are questions that can potentially be investigated in future research.
• Irrespective of questioned-speaker condition, and despite some statistical noise in the pattern of results, there was a clear exponential relationship between questioned-speaker net-speech duration and *C*_llr_: From 5 s to 30 s there was a rapid decline in *C*_llr_ values, after which the rate of decline slowed, and by 60s for male speakers and 90 s for female speakers they had asymptoted. This result can inform expectations for performance in future casework, and also suggests that for future data collection it may not be necessary to collect questioned-speaker-condition recordings that are longer than 90 s net speech.^12^
• Across male and female speakers, and across different durations of questioned-speaker recordings, the order of which condition gave best, second best, and worst results was not consistent. The questioned-speaker condition that had no mismatch with the known-speaker condition (μ-law + G.723.1) did not consistently result in lower *C*_llr_ values than the other conditions. One could conclude that, in general, the differences in *C*_llr_ values between the different conditions were relatively small. The biggest differences tended to occur for shorter durations (15 s or less), which one would expect to be more susceptible to statistical noise.

**Fig. 8:**
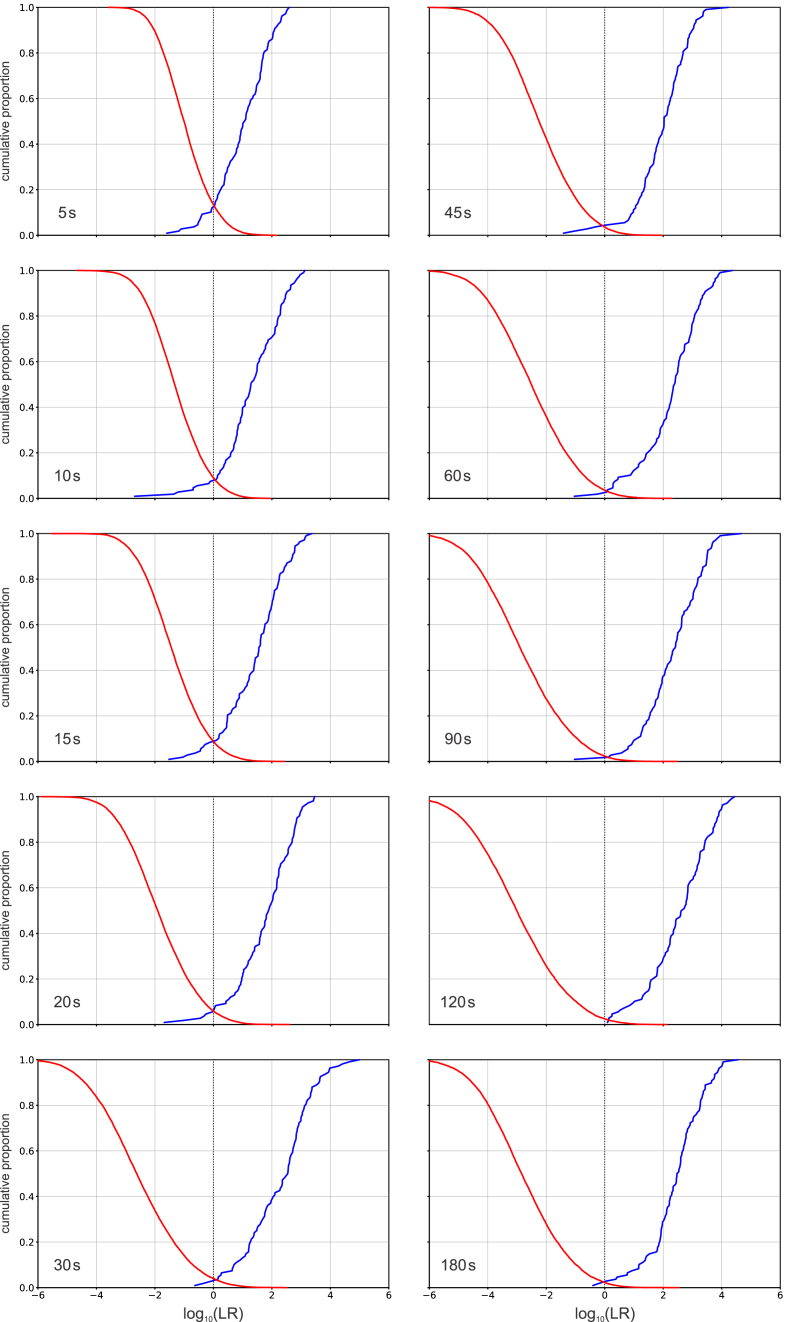
Tippett plots of the results of validating E^3^FS^3^α using case-specific data (female Australian-English speakers and questioned-speaker condition μ-law + G.729a) at different questioned-speaker net-speech durations.

Fig. 8 presents a set of Tippett plots for female speakers and questioned-speaker condition μ-law + G.729a. The patterns of results for other combinations of population and questioned-speaker condition were broadly similar. At all different questioned-speaker net-speech durations, the results were well calibrated – the same-speaker and different-speaker curves crossed near a log-likelihood-ratio value of 0. Even for the shortest questioned-speaker net-speech duration (5 s), the Tippett plot indicates that the validation results would support likelihood-ratio values into the hundreds in favour of either the same-speaker hypothesis or the different-speaker hypothesis (log_10_ likelihood ratios beyond +2 and −2 respectively), but not values far beyond these ranges (see [4] §2.11). For questioned-speaker net-speech durations of 30 s or more, the range extended into the thousands in favour of the same-speaker hypothesis and the tens of thousands in favour of the different-speaker hypothesis (log_10_ likelihood ratios beyond +3 and −4 respectively). Asymmetries of this type are often observed in the results of validations of forensic-voice-comparison systems, but the large negative log likelihood ratios may correspond to pairs of speakers who sound quite different from one another and are therefore unlikely to be submitted for forensic comparison.

## 6 General discussion and conclusion

In the context of the case: We were happy with the results of the benchmark validation of E^3^FS^3^α using the *forensic_eval_01* data – performance was equal to that of the best-performing system with previously published results validated on this dataset. We therefore proceeded with validations using case-specific recording conditions and durations, but using recordings of male speakers rather than the case-specific female speakers. We were again happy with the validation results. We therefore proceeded with fully-case-specific validations using recordings of female Australian-English speakers. We were again happy with the validation results, and we included the latter set of validation results in the casework report. The report also documented that our validation procedures followed the recommendations in the *Consensus on validation of forensic voice comparison* [4] – a checklist was developed, and a second practitioner checked whether the report written by the first practitioner described whether, and if so how, each recommendation had been followed.^13^ We then proceeded to use E^3^FS^3^α to compare the actual questioned-speaker and known-speaker recordings in the case, and to report the resulting likelihood-ratio values. The reported likelihood-ratio values were supported by the validation results. Usually, in the context of a case, we would only conduct a fully-case-specific validation and report the results. Because E^3^FS^3^α was a new system that had not been previously validated, we conducted the other validations first.

The questioned-speaker net-speech durations used for the case-specific validations reported in the present paper did not match the exact durations of the questioned-speaker recordings in the case, but covered a range of durations providing results that are potentially informative for expectations regarding performance in future casework, and potentially informative for future data-collection plans. E^3^FS^3^α performance asymptoted by 90 s questioned-speaker net speech. This suggests that for future data collection it may not be necessary to collect questioned-speaker-condition recordings that are longer than 90 s net speech. Note, however, that this is 90 s worth of extracted feature vectors. In order to be able to extract this number of feature vectors the recordings will usually have to be substantially longer, e.g., for a conversation involving two people, in order to have a high probability of being able to extract at least this many feature vectors from each speaker, we would recommend making a 5-min-long recording. It may be more useful to make more 5-min-long recordings in more different conditions than to make fewer longer recordings in fewer conditions.

E^3^FS3α is the first working version (an alpha version) of the E^3^FS^3^ core software tools. We will continue to develop E^3^FS^3^ overall, including core software tools. In due course, a beta version of the software tools will be released to our partner organizations for field testing by their operational forensic laboratories. After revisions informed by field testing, we plan to produce the first general release (some components that can stand alone may be released earlier than others). E^3^FS^3^ core software tools are designed to be flexible and provide the user with various options and the ability to retrain models. We plan to explore different options and additions, and to use different datasets for training the x-vector extractor and as out-of-domain CORAL data (or CORAL+ data), which may lead to improved performance. This may include exploring whether performance on female speaker can be improved to match the performance on male speakers.

The present paper has provided an example of validation of a forensic-voice-comparison system under casework conditions. We hope that that this example can be copied in both casework practice and in future research aimed at informing casework practice.

## Disclaimer

All opinions expressed in the present paper are those of the authors, and, unless explicitly stated otherwise, should not be construed as representing the policies or positions of any organizations with which the authors are associated.

## Author contributions

**Philip Weber:** Methodology, Software, Validation, Investigation, Writing - Original Draft, Writing - Review & Editing.

**Ewald Enzinger:** Methodology, Software, Writing - Review & Editing.

**Beltrán Labrador:** Methodology, Software, Writing - Review & Editing.

**Alicia Lozano-Díez:** Methodology, Writing - Review & Editing.

**Daniel Ramos:** Methodology, Writing - Review & Editing, Supervision.

**Joaquín González-Rodríguez:** Methodology, Writing - Review & Editing, Supervision.

**Geoffrey Stewart Morrison:** Conceptualization, Methodology, Validation, Investigation, Writing - Original Draft, Writing - Review & Editing, Visualization, Supervision, Funding acquisition.

## Declaration of competing interest

Dr Morrison is the Director of Forensic Evaluation Ltd, and Dr Weber and Dr Enzinger have worked for Forensic Evaluation Ltd on a contract basis.

## Footnotes

1 https://www.sciencedirect.com/journal/speech-communication/special-issue/10KTJHC7HNM.

2 Principles for the design of Python, “The Zen of Python”, are listed at https://www.python.org/dev/peps/pep-0020/.

3 The forensic voice comparison for this case was performed by Forensic Evaluation Ltd.

4 Note that scores output by PLDA are uncalibrated likelihood ratios, they are not similarity-only scores [[30], [31], [32]].

5 Each pseudo-data point is weighted by $w^{\psi }=\kappa^{\psi }/2N$, where $\kappa^{\psi }$ is the number of pseudo-speakers ($\kappa^{\psi }=1$) and N is the number of speakers used to train the model. See [36] for further explanation.

6 FFmpeg version 4.3.1-2020-11-19-full_build-www.gyan.dev. http://ffmpeg.org/.

7 MediaInfo version 20.09. https://mediaarea.net/en/MediaInfo.

8 Audacity version 2.4.2. https://www.audacityteam.org/.

9 In the descriptions of case-specific validations “net speech” refers to the duration equivalent to the number of feature vectors extracted. 1 s equals 100 feature vectors.

10 The arrows indicate the transformation of the audio recording from one format to another.

11 This is similar to the behaviour of the standard error of the mean compared to the sample standard deviation.

13 A version of this checklist is available at http://e3fs3.forensic-voice-comparison.net/.

## Footnotes

12 We say more about this in the general discussion and conclusion section, §5.
